# Sandwich-structured poly(vinylidene fluoride-hexafluoropropylene) composite film containing a boron nitride nanosheet interlayer†

**DOI:** 10.1039/c9ra09780e

**Published:** 2020-01-13

**Authors:** Fujia Chen, Yujiu Zhou, Jimin Guo, Song Sun, Yuetao Zhao, Yajie Yang, Jianhua Xu

**Affiliations:** State Key Laboratory of Electronic Thin Films and Integrated Devices, School of Optoelectronic Science and Engineering, University of Electronic Science and Technology of China Chengdu 610054 China jianhuaxu215@163.com +86-28-83206123 +86-28-83207027; School of Electronics and Information, Jiangsu University of Science and Technology Zhenjiang 212003 China zhaoyuetao@yeah.net

## Abstract

High performance dielectric polymer materials are a key point for energy storage capacitors, especially film capacitors. In this paper, a sandwich-structured polymer film is constructed to achieve high energy density and high efficiency. High dielectric materials of poly(vinylidene fluoride-hexafluoropropylene) (P(VDF-HFP)) doped with barium titanate (BaTiO_3_) are used as the outer layer to achieve a high dielectric constant, and a boron nitride nanosheet (BNNS) layer is inserted between P(VDF-HFP)/BaTiO_3_ to obtain a high breakdown field strength of composite films. The results indicate that when the doping amount of the BaTiO_3_ nanoparticles reaches 10 wt% and the mass fraction of the BNNS layer is 0.75 wt%, a significant improvement of energy storage performance is obtained. The energy storage density of the P(VDF-HFP)/BaTiO_3_/BNNSs composite film can reach 8.37 J cm^−3^, which is higher than 6.65 J cm^−3^ of the pure P(VDF-HFP) film. Compared with the P(VDF-HFP) film doped with BaTiO_3_, significant improvement of the breakdown field strength (about 148.5%) is achieved and the energy storage density increases 235% accordingly, resulting from the inserted BNNSs layer blocking the growth of electrical branches and suppressing leakage current. This novel sandwich-structured film shows promising future applications for high performance dielectric capacitors.

## Abstract 2

A novel composite material formed by adding high dielectric inorganic ceramic particles and BNNSs interlayer into a sandwich-structured film.

## Graphical abstract

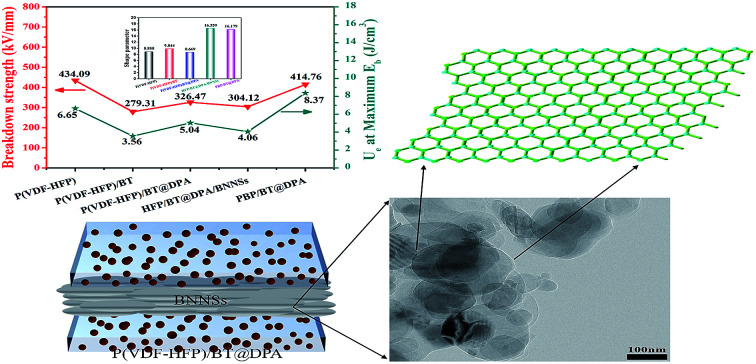

## Introduction

Dielectric polymer materials such as poly(vinylidene fluoride-hexafluoropropylene) (P(VDF-HFP)) are particularly widely used in energy storage, especially film capacitors, because of their high dielectric constant, low loss, and ease of processing. Electrostatic film capacitors have been widely used in electric vehicles, flexible high-voltage DC power transmission and transformation systems, medical equipment, electromagnetic ejection systems and other fields.^1–4^ However, the energy storage density of the most polymer materials is generally low, which limits the development of film capacitors. For instance, the energy storage density of biaxially oriented polypropylene (BOPP) film is only 1 J cm^−3^.^5^ Energy storage density is a key indicator for evaluating the performance of film capacitors. As a key material for film capacitors, dielectric films can be divided into linear polymers and nonlinear polymers.^6^ The storage density of linear polymers can calculate by the formula:
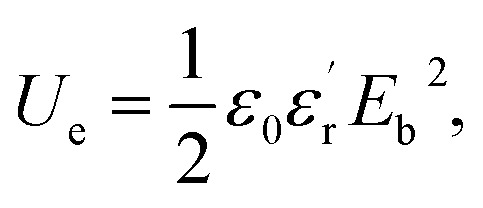
and the energy storage density of a nonlinear polymer can be calculated according to the formula:
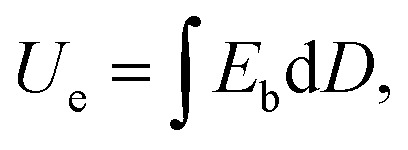
where *E*_b_ is the breakdown field strength and *D* is the electrical displacement which can be expressed as 
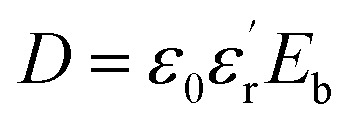
 (*ε*_0_ is the vacuum dielectric constant, and 
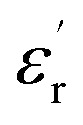
 is the relative dielectric constant of the material). Therefore, it is believed that the dielectric constant and breakdown field strength of the lifted material can effectively increase the energy storage density.

In general, compared with polymer materials, inorganic ceramic materials exhibit higher dielectric constant. In recent years, composite materials composed of polymer materials and inorganic ceramics have been widely studied.^7–10^ In order to increase the dielectric constant of polymer materials, the introduction of inorganic ceramic particles, such as BaTiO_3_ (BT), Ba_*x*_Sr_1−*x*_TiO_3_ (BST), P(Mg_1/3_Nb_2/3_)O_3_–PbTiO_3_, *etc.*,^11–14^ into polymer matrix is considered an effective method. However, in order to achieve a higher dielectric constant of the composite material, it is generally necessary to add a higher volume fraction of the ceramic materials, which leads to problems with the compatibility and dispersion of ceramic particles with the matrix. Hence, several kinds of research have been carried out to solve this problem: surface modification of ceramic fillers,^15–17^ regulation of filler distribution,^18,19^ the addition of third phase fillers, *etc.*^20–22^ Ceramic materials after surface treatment and structural control have better compatibility and dispersion.

Boron nitride nanosheets (BNNSs) are a typical two-dimension nanomaterial that have become an ideal filler for high performance polymer composites. It has a high forbidden bandwidth (≈6 eV) and a high intrinsic breakdown voltage.^23,24^ It is widely used in research to improve the energy storage characteristics of polymer materials. For example, BNNSs are directly added to the matrix, which resulted in a significantly improved breakdown field strength.^21,25,26^ Most of these work is centered around the direct compounding of fillers and polymer materials, as well as the uniform distribution of fillers in polymer composite films. However, the structural design of film is rarely studied. Recently, Huang *et al.* used the structural design of BNNSs insulation layer to improve the breakdown field strength and reduce the loss.^27^ This structure has been proven very reliable for increasing energy storage density. In order to improve the energy storage characteristics of materials, BaTiO_3_ nanoparticles were chosen as the ceramic filler to increase the dielectric constant of the matrix. At the same time, considering that BaTiO_3_ will cause a decrease in breakdown field strength, inspired by their work,^27^ BNNSs were chosen as the third phase filler and designed as a separate mezzanine. While using the inorganic ceramic particles to increase the dielectric constant, the effect of suppressing the reduction of breakdown field strength is achieved by adding BNNSs. Through the coordination between the materials of each phase, it should be an effective way to increase the dielectric constant while suppressing a significant reduction in breakdown strength. As for details, P(VDF-HFP) is selected and a sandwich structure polymer films are constructed. High dielectric materials of P(VDF-HFP) doped with BaTiO_3_ are used as the outer layer, and a BNNSs layer is inserted between P(VDF-HFP)/BaTiO_3_. Different from the method of directly doping BNNSs into the matrix material, the separate mezzanine is prepared by solution casting. Each layer structure is sequentially prepared on the quartz substrate by a scraper from bottom to top, finally forming a sandwich composite film. In addition, BaTiO_3_ nanoparticles are surface-modified with dopamine to improve the dispersion of nanoparticles in the matrix. The sandwich-structured film presents a significant improvement in dielectric constant and energy storage density, the breakdown field strength of the composite film is not seriously reduced by the addition of BaTiO_3_. For instance, the sandwich-structured film has a dielectric constant of 10.99, which is 1.446 times of pure P(VDF-HFP), and the energy storage density has also improved significantly. This work provides a new way to obtain high performance energy storage media composites.

## Materials and methods

P(VDF-HFP) was purchased from Sigma-Aldrich (Solef 427160). *N*,*N*-Dimethylformamide (DMF), isopropanol, ammonia and absolute ethanol were purchased from Chengdu Kelong chemical reagent factory. BaTiO_3_ was purchased from Sigma-Aldrich (B118840, <100 nm). Dopamine hydrochloride was purchased from MERYER (M21851). Hexagonal boron nitride (h-BN) was purchased from Sigma-Aldrich (B106033). All chemicals were used as received.

First of all, ultrasonic degradation method which is reported previously is applied here to prepare BNNSs.^21,26,28^ 2 g h-BN was added to 200 mL DMF solvent, continuously stripping by ultrasonic breaker for 20 h (340 W). After the completion of the ultrasonic, the solution was placed in a centrifuge and centrifuged at 3000 rpm for 45 min, then the supernatant was collected and centrifuged again at 9000 rpm for 10 min. In order to ensure the dispersion of BNNSs, freeze-drying method was used to dry the precipitate at the bottom of the centrifuge tube. As reported in the literature, BNNSs were prepared by ultrasonic degradation method with a yield of 2.5–3%.^27,29^ The experimentally collected BNNSs were 60 mg, and the acquisition rate was within the normal range. The experimental results preliminary indicated that h-BN was stripped successfully.

In addition, barium titanate nanoparticles was coated by dopamine through water bath.^30^ 0.04 g of dopamine hydrochloride was dissolved in an aqueous ammonia solution with pH of 8.5 to form a mixed solution of 2 g L^−1^. Appropriate amount of BaTiO_3_ nanoparticles was added to the above solution, and stirred in a water bath at 60 °C for 12 h. The product was collected by centrifugation. Finally, BT@DPA particles were vacuum dried at 60 °C for 10 h.

**Fig. 1:**
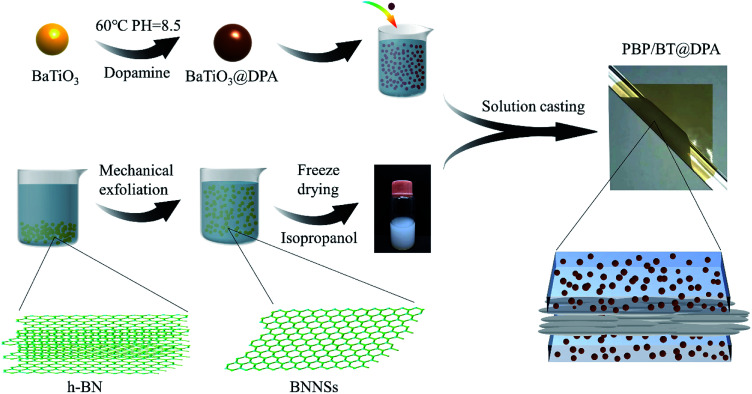
Experimental flow chart: surface modification of BaTiO_3_ by dopamine, preparation of BNNSs and fabrication of sandwich structure film.

The specific experimental process can be seen in Fig. 1. Pure P(VDF-HFP) was first physically blended with 10 wt% of BT@DPA nanoparticles in DMF solvent and mechanically stirred for 12 h until P(VDF-HFP) was completely dissolved. The BNNSs were dispersed in isopropanol to form a solution with 0.75 wt%. The preparation process of the sandwich-structured film was as follows. Firstly, a P(VDF-HFP)/BT@DPA bottom layer film was casted on a quartz substrate with a scraper, dried at 70 °C for 6 hours in a vacuum oven to remove solvents. Then, the interlayer of BNNSs and the top layer film were casted through the same process. The sandwich-structured film was dried overnight in a vacuum oven at 70 °C to remove the solvents. Finally, the quartz substrate was placed on a heating table at 200 °C for 10 min and quenched in ice water. Hereinafter, this sandwich-structured film is named PBP/BT@DPA. In addition, a solution of only doped with BaTiO_3_ nanoparticles and a solution of co-doped with BaTiO_3_ nanoparticles and BNNSs (the doping amount of BNNSs and BaTiO_3_ were 10 wt%) were prepared. The same preparation process was used to prepare four other films: pure P(VDF-HFP), P(VDF-HFP)/BT, P(VDF-HFP)/BT@DPA, HFP/BT@DPA/BNNSs, all of which were single layer (the thickness were 12–13 μm).

A series of morphological characterization and electrical performance tests on the prepared materials and films were performed. First of all, BNNSs were characterized by Atomic Force Microscope (AFM) and Transmission Electron Microscope (TEM) respectively. The cross section of the films were observed by Scanning Electron Microscopy (SEM, Hitachi S-4800). In addition, the films were characterized by Fourier-transform infrared spectroscopy (FTIR, 8400S, Shimadzu) and X-ray diffraction (XRD, Malvern Panalytical, Inc.). The thickness of film was measured by a magnetic induction thickness-meter (Fischer DUALSCOPE MPO). Finally, dielectric spectrum characteristics were measured by using a precision impedance analyzer (4294A, Agilent Technologies, Inc.) from 40 Hz to 5 MHz. Breakdown field strength was tested with a withstand voltage tester (TH9120, Changzhou Tonghui electronics co., LTD), and *P*–*E* hysteresis loops of films were measured by using a ferroelectric tester (Radiant Technologies, Inc.).

## Results & discussion

### Characterization of BNNSs and films

**Fig. 2:**
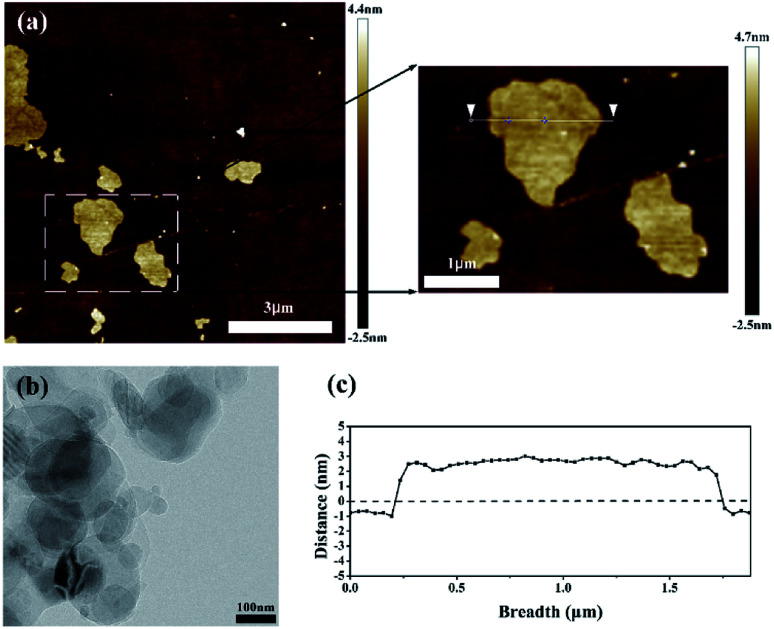
Micromorphology of BNNSs: (a) AFM image, (b) TEM characterization images and (c) the thickness of a piece of BNNSs intercepted.

**Fig. 3:**
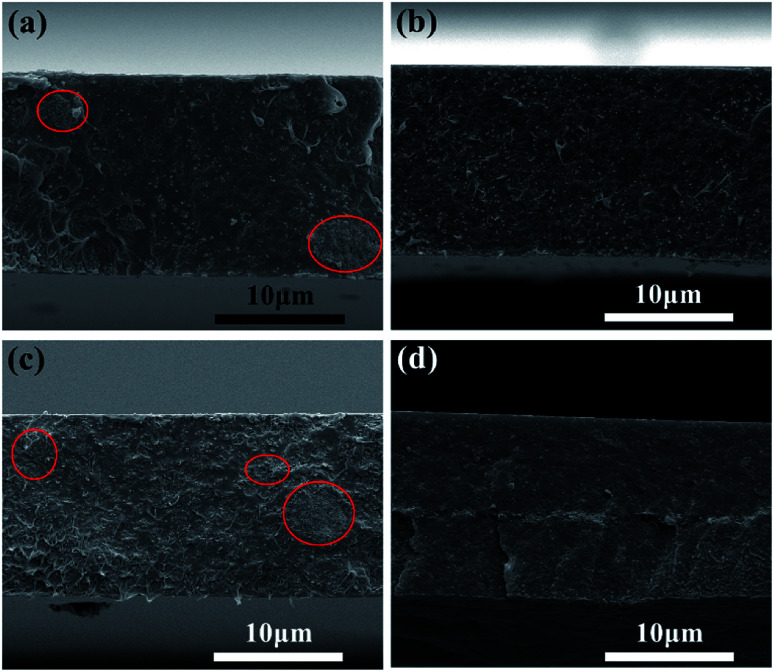
SEM images of film cross section: (a) P(VDF-HFP)/BT, (b) P(VDF-HFP)/BT@DPA, (c) HFP/BT@DPA/BNNSs and (d) PBP/BT@DPA.

The AFM image is shown in Fig. 2(a) and the degree of peeling and aggregation of BNNSs are expressed by TEM as shown in Fig. 2(b). The picture shows that h-BN after stripping has become nanosheets of different sizes, and there is no significant agglomeration between the BNNSs. Fig. 2(c) shows a cross-sectional height map of a piece of nanosheet randomly taken, indicating that the thickness of BNNSs is around 2–3 nm. These results verify that h-BN is successfully stripped into BNNSs with very low thickness and relatively good dispersion state. The microscopic morphology of the cross-section of the films was analyzed by SEM. In order to protect the cross-section morphology of films, all characterization samples were prepared in liquid nitrogen and then sputter-coated with a homogeneous gold layer. At a magnification of 10000×, the internal morphology of films can be clearly observed. The mixed doped film filler exhibits a poor distribution effect as shown in Fig. 3(c), while the dispersibility of the dopamine-coated BaTiO_3_ particles was significantly improved as shown in Fig. 3(a) and (b). When two nanomaterials are simultaneously doped into the matrix, not only the aggregation between the single particles but also the mutual adsorption between the two phases occur. Such a morphology will lead to a significant increase in dielectric loss, as evidenced by subsequent testing of the film. It can be seen from Fig. 3(d) that the upper and lower layers are matrix films doped with BaTiO_3_ particles, and the filler is evenly distributed among them. There is a thin intermediate layer in the middle, namely the cast BNNSs insulation layer, and the nanosheets are densely arranged in the matrix. Three layers can be clearly observed, which verifies that this is indeed a film of sandwich structure.

FTIR and XRD results are visible in the ESI.† FTIR technology is a productive method to identify the different crystal phases of PVDF-based polymers, and the spectra of films are demonstrated in Fig. S1.† P(VDF-HFP) is a polar polymer with a long molecular chain extending from the crystalline region to the amorphous region. The absorption peaks at 879 cm^−1^ are the amorphous phase absorption peak of P(VDF-HFP), while the absorption peaks at 764 cm^−1^ and 796 cm^−1^ are the α phase absorption peaks of P(VDF-HFP) film.^31,32^ These films mainly exhibited α phase, indicating that the preparation process used had no significant effect on the crystalline phase of the matrix material. Analyzing the XRD results in Fig. S2,† the characteristic diffraction peaks of BaTiO_3_ are very obvious, and the diffraction peaks of BaTiO_3_ at (100), (110), (111) crystal planes can be clearly seen.^33,34^ It is worth noting that a diffraction peak appears at 2*θ* ≈ 26.4°, corresponding to the BNNSs (002) crystal plane.^21,35^ Because the amount of BNNSs added to the composite film is small, the peak intensity is very weak due to the influence of the BaTiO_3_ diffraction peak.

### Dielectric performances of P(VDF-HFP) films

**Fig. 4:**
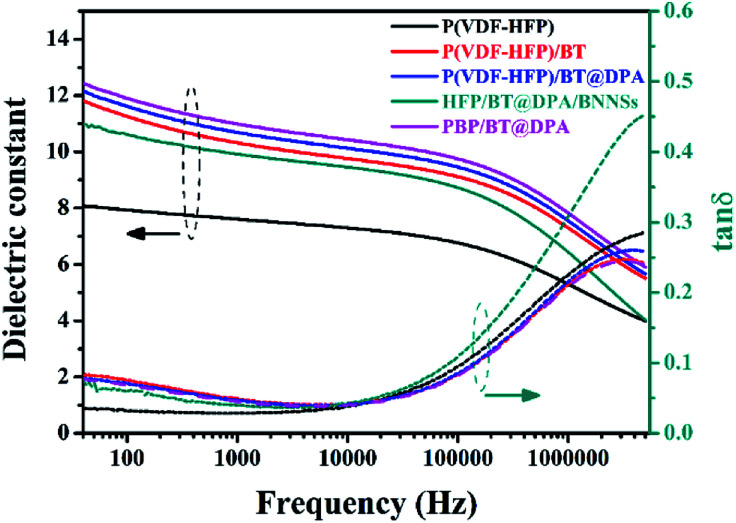
Dielectric spectrogram: relationship between dielectric constant 
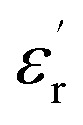
 and dielectric loss (tan *δ*) as a function of frequency.

The dielectric spectrum of P(VDF-HFP) films are shown in Fig. 4. The dielectric constant 
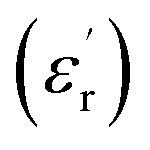
 decreases with increasing frequency, due to the periodic transformation of the electric field at high frequency resulting in the decrease of charge distribution in the direction of the electric field. In other words, the role of the molecular dipole moment is gradually reduced. It can be seen from the figure that the dielectric constant of the composite film doped with BaTiO_3_ is much higher than that of the pure P(VDF-HFP) film. The 
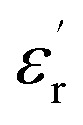
 of PBP/BT@DPA at 1 kHz is 10.99, while the pure P(VDF-HFP) film is only 7.6, which shows a very large enhancement rate of 
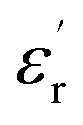
 (144.6%). In contrast, the 
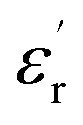
 of HFP/BT@DPA/BNNSs is not increase significantly, because the doping amount of BNNSs is 10 wt% and its dielectric constant is inherently low (∼4). The result shows that the effect of BaTiO_3_ on the improvement of dielectric constant is very obvious. The dielectric loss tan *δ* of all films shows a gradually rising trend.^36^ Compared with pure P(VDF-HFP) film, the composite film doped with BaTiO_3_ particles shows a higher loss at low frequency. On the one hand, Maxwell–Wagner–Sillars (MWS) polarization dominates the dielectric response at low frequencies. On the other hand, the addition of BaTiO_3_ particles also increases the dielectric loss.^37,38^ The loss increases rapidly at high frequencies, which can be attributed to the dynamic relaxation of the matrix material.^39^ The dielectric loss of HFP/BT@DPA/BNNSs film is much larger than other films, this data validates the results of the SEM. The dielectric loss of the polymer is not only related to the nature of the material itself, but also to the state of aggregation between the phases.^40,41^ The agglomeration and local accumulation of ceramic particles directly increase the porosity of the mixture and increase the water absorption of the composite. When there are many pores in the matrix, the phase composition of each material in the actual matrix deviates from the theoretical value, and the dielectric loss increases by order of magnitude. The moisture absorption of pores will increase the moisture content of the dielectric material, which has a great influence on the dielectric loss. The insulation layer of BNNSs are separately added into sandwich-structured film, avoiding mutual aggregation between the phases, which reduces the porosity of the composite and achieves better interface conditions. Compared with the film directly doped with two kinds of inorganic materials, this structure makes the density distribution of the film more uniform, which is very helpful for improving the dispersion of the filler. However, the insulation layer of BNNSs doesn't significantly reduce the dielectric loss at high frequency. This may be due to the large amount of BaTiO_3_ added, and the effect of suppressing the loss of BNNSs is not conspicuous. Ceramic fillers can effectively increase the dielectric constant, but the content is often high, which also weakens the flexibility of the polymer film. Compared with PVDF, P(VDF-HFP) has a lower glass transition temperature and a higher degree of amorphization, resulting in abetter flexibility. This is the reason why P(VDF-HFP) is chosen as the matrix material.^42,43^ In short, the composite film with BNNSs interlayer shows a great improvement in dielectric properties. Not only does the dielectric constant increases significantly, but also the dielectric loss is suppressed to some extent.

### Breakdown characteristics of several films

**Fig. 5:**
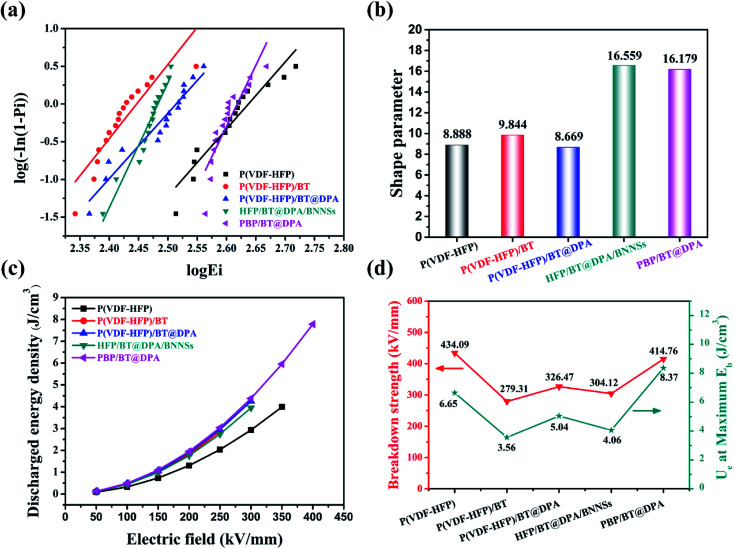
(a) Weibull distribution, (b) shape factor *β*, (c) storage density of films under different electric field strengths and (d) the storage density of the films under the maximum breakdown field strength.

The Weibull distribution is used to evaluate the breakdown field strength characteristics, the expression is as follows:*P*(*E*) = 1 − exp((−*E*/*E*_b_)^*β*^),where *P*(*E*) is the cumulative electrical breakdown probability, *E* is the experimental electrical breakdown strength, and *E*_b_ is the characteristic breakdown strength when the cumulative probability reaches 63.2%. The shape factor *β* shows the degree of dispersion of the data, and the higher the value, the higher the reliability. Before conducting the comparative experiment, the BNNSs concentration test was performed to choose a suitable concentration. Using isopropyl alcohol as a solvent, BNNSs were formulated into a solution at a ratio of 0.25 wt%, 0.5 wt%, 0.75 wt% and 1 wt%. Four solutions were added as an insulating layer to the matrix material and tested for their breakdown field strength. The results are visible in Fig. S3 of the ESI.† After 0.75 wt% of BNNSs were added into the matrix, the breakdown strength reached 414.76 kV mm^−1^, the highest of all samples. Therefore, the 0.75 wt% of BNNSs was selected for subsequent experiments, and the test results of several films are shown in Fig. 5(a) and (b). The *E*_b_ and *β* of pure P(VDF-HFP) film are 434.09 kV mm^−1^ and 8.88, respectively. Compared with the P(VDF-HFP) film, the breakdown field strength of the film doped only with BaTiO_3_ particles is seriously reduced. The breakdown field strength of a three-phase mixed doped film with a large amount of BNNSs added is not improved, which is at the same level as P(VDF-HFP)/BT@DPA. After three-phase mixed, there are many defects and holes inside the film inevitably. As a kind of pre-breakdown phenomenon in dielectric film, electric branches mainly occur in areas where defects, voids, and conductive fillers are concentrated. The three-phase mixed film, despite the large content of BNNSs, does not form a complete topological barrier, and the electric branches will grow freely in the voids, which is also the direct cause of the decrease in breakdown field strength. On the contrary, during the growth of electric tree branches, the BNNSs interlayer acts as a barrier or scattering interface in sandwich-structured film.^27^ The breakdown field strength of PBP/BT@DPA is 414.76 kV mm^−1^, which is not much different from P(VDF-HFP). The shape factor *β* of the films doped with BNNSs is higher than other samples, in particular, the shape factor *β* of PBP/BT@DPA is 1.82 times that of pure P(VDF-HFP). The results indicate that the introduction of BNNSs interlayer can significantly improve the breakdown characteristics of composites compared to directly doped BaTiO_3_ nanoparticles.

### Energy storage characteristics of several P(VDF-HFP) films

The composite film prepared by referring to the structural design of the BNNSs insulating layer is to increase the dielectric constant under the condition of maintaining the breakdown field strength, thereby increasing the energy storage density of the film. From the results, the purpose of experiment are also verified. As shown in Fig. 5(c), the energy storage density of modified films are higher than of pure P(VDF-HFP) film under the same breakdown field strength. The energy storage density of P(VDF-HFP)/BT, P(VDF-HFP)/BT@DPA, and HFP/BT@DPA/BNNSs is close to the limiting value at 300 kV mm^−1^, however, the energy storage density of PBP/BT@DPA can continue to rise. The energy storage density of the film under the maximum breakdown field strength are calculated, and the results are shown in Fig. 5(d). The pure P(VDF-HFP) film has a storage density of 6.65 J cm^−3^ under the maximum breakdown field strength. In contrast, compared with P(VDF-HFP), the films of P(VDF-HFP)/BT, P(VDF-HFP)/BT@DPA, and HFP/BT@DPA/BNNSs, not only the breakdown field strength is lower, but the energy storage density is also not as good as the former. The sandwich-structured film exhibits a storage density of 8.37 J cm^−3^ under the maximum breakdown field strength (414.76 kV mm^−1^), which is 125.9% higher than that of pure P(VDF-HFP) film. The performance of the prepared PBP/BT@DPA composite film is compared with other polymer films reported in the literature, as shown in Table 1. The table reveals that the sandwich-structured film of this work has better comprehensive properties than other films. In addition, there is still a lot of research space for this sandwich-structured film, and how to further improve the breakdown field strength is very worth exploring in subsequent studies.

**Table 1:** Comparison of dielectric constant, electric field and energy density

Materials	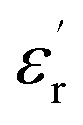 (1 kHz)	*E*_b_ (kV mm^−1^)	*U*_e_ (J cm^−3^)	References
PBP/BT@DPA	10.99	414.76	8.37	This work
Polypropylene (PP)	2.2	640	1–1.2	5
Polyester (PET)	3.3	570	1–1.5	5
Polycarbonate (PC)	2.8	528	0.5–1	5
PVDF	8	400	2.8	44
PVDF/BST-NPs	36	250	3.9	45
8 wt% BNNSs/PVDF	8.3	486	7.25	46
0.5 wt% BaTiO_3_@BNNSs/PVDF	12.5	350	7	47
7 vol% Fe_3_O_4_@BNNSs/PVDF	16	300	7.1	21
7.5 vol% F–TiO_2_/P(VDF-HFP)	12	160	1.4	48
5 vol% BaTiO_3_@PMPCS/P(VDF-HFP)	20	300	7.5	49
0.4 wt% BNNSs/P(VDF-CTFE)	23	300	6.8	50

**Fig. 6:**
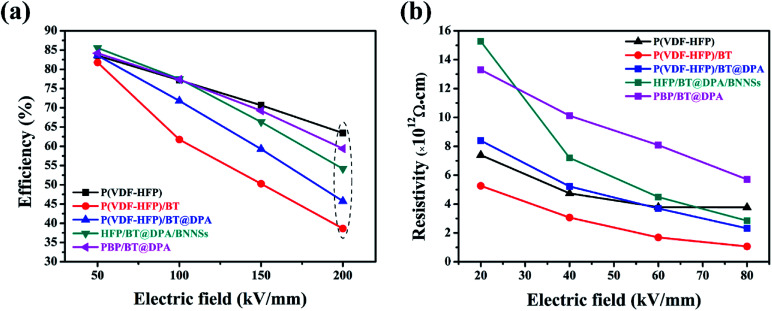
(a) Charge–discharge efficiency and (b) insulation resistivity.

*P*–*E* hysteresis loops and leakage currents of several films are visible in the ESI,† and the charge–discharge efficiency are shown in Fig. 6(a). The efficiency of the film doped with BaTiO_3_ decreases very sharply with the increase of electric field, and the charge–discharge efficiency is less than 50% at 200 kV mm^−1^. Compared with P(VDF-HFP), the efficiency of the sandwich-structured film does not decrease significantly. As the electric field strength increases, the difference in electrical properties between the base material and the filler causes an increase in the charge carrier concentration of the film, thereby causing a decrease in charge–discharge efficiency during the electric field switching process. BNNSs have a high length height ratio and specific surface area, and the high energy barrier of the nanosheets can suppress the movement of charges under high electric fields.^51^ The interlayer formed by solution casting has a dense network structure and plays a vital role in hindering the migration of charge carrier.^52^ In Fig. 6(b), the insulation resistivity of the film was calculated according to leakage currents. The leakage current of the dielectric film can be expressed as:*I*_L_ = *U*/*R*_I_,where *U* is an applied voltage and *R*_I_ is an insulation resistance. The *R*_I_ can be calculated by other parameters:*R*_I_ = *ρ*_I_*d*/*A*,where *ρ*_I_ is insulation resistivity, the *d* is the dielectric film thickness, and *A* is the area of the aluminum electrode evaporated on the dielectric film. From these two equations, the calculation formula of the insulation resistivity can be derived:
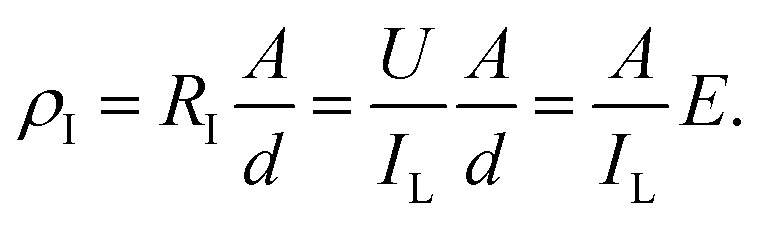


Under low electric field, the conductive effect of BaTiO_3_ is not obvious, so the HFP/BT@DPA/BNNSs film with 10 wt% BNNSs added shows the highest insulation resistivity. As the electric field strength increases slowly, the conduction of BaTiO_3_ is also gradually enhanced. Meanwhile, the role of the BNNSs interlayer is gradually becoming prominent, which hinders the conduction of leakage current. Under the same electric field strength, sandwich structure film has a higher insulation resistivity.

## Conclusion

A novel composite material formed by adding high dielectric inorganic ceramic particles and BNNSs interlayer into a sandwich-structured film. This method is indeed feasible by increasing the dielectric constant to achieve high storage density while ensuring a high breakdown field strength. The doped BaTiO_3_ particles are as high as 10 wt%, while the added BNNSs insulating layer is only 0.75 wt%. Compared with the direct doping of a large amount of BNNSs, this method greatly reduces the amount of BNNSs used. The dielectric constant of the composite film is 10.99, which is 144.6% of that of the pure P(VDF-HFP) film. The breakdown field strength is not much different from pure P(VDF-HFP), furthermore, the shape factor is greatly improved. The sandwich-structured film with BNNSs interlayer not only increases the energy storage density, but also reduce the leakage current conduction with the BNNSs interlayer as a topological barrier, keeping the charge–discharge efficiency of the film at a normal level. In short, this is a reliable sandwich structure that provides new ideas for simultaneously increasing the dielectric constant and breakdown field strength. In future work, how to increase the breakdown field strength while maintaining a high dielectric constant and charge–discharge efficiency may be the focus of research.

## Conflicts of interest

There are no conflicts to declare.

## Supplementary Material

RA-010-C9RA09780E-s001
