# Supplementary material for: Sandwich-structured poly(vinylidene fluoride-hexafluoropropylene) composite film containing a boron nitride nanosheet interlayer
Source: RSC Adv. 2020 Jan 13;10(4):2295–302. doi: 10.1039/c9ra09780e (PMC9048770; doi:10.1039/c9ra09780e)
Supplement: RA-010-C9RA09780E-s001 [file RA-010-C9RA09780E-s001.pdf]

Electronic Supplementary Material (ESI) for RSC Advances.
This journal is © The Royal Society of Chemistry 2019

Supplementary materials

Sandwich-structured P(VDF-HFP) composite film incorporated with boron nitride nanosheets interlayer

Fujia Chen[1], Yujiu Zhou[1], Jimin Guo[1], Song Sun[1], Yuetao Zhao[2,†], Yajie Yang[1] and Jianhua Xu[1,‡]

1 State Key Laboratory of Electronic Thin Films and Integrated Devices, School of Optoelectronic Science and Engineering, University of Electronic Science and Technology of China, Chengdu 610054, China.

2 School of Electronics and Information, Jiangsu University of Science and Technology, Zhenjiang 212003, China.

* Correspondence: zhaoyuetao@yeah.net (†); jianhuaxu215@163.com (‡).

Tel.: +86-28-83207027

Fax: +86-28-83206123

1. FTIR

The prepared experimental samples were characterized by FTIR.

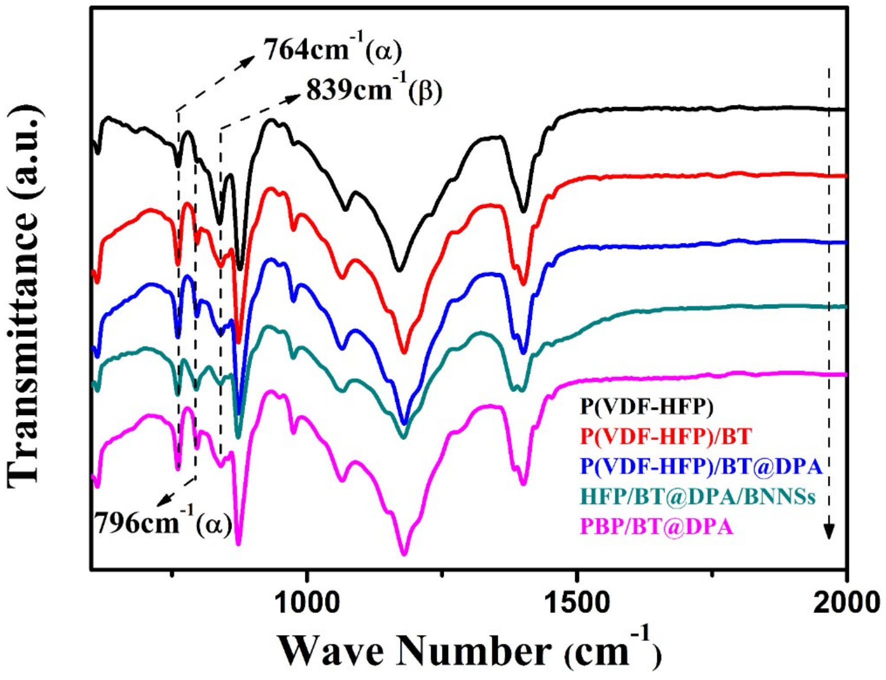

Figure S1 FTIR image of several P(VDF-HFP) films

2. XRD

The prepared experimental samples were characterized by XRD.

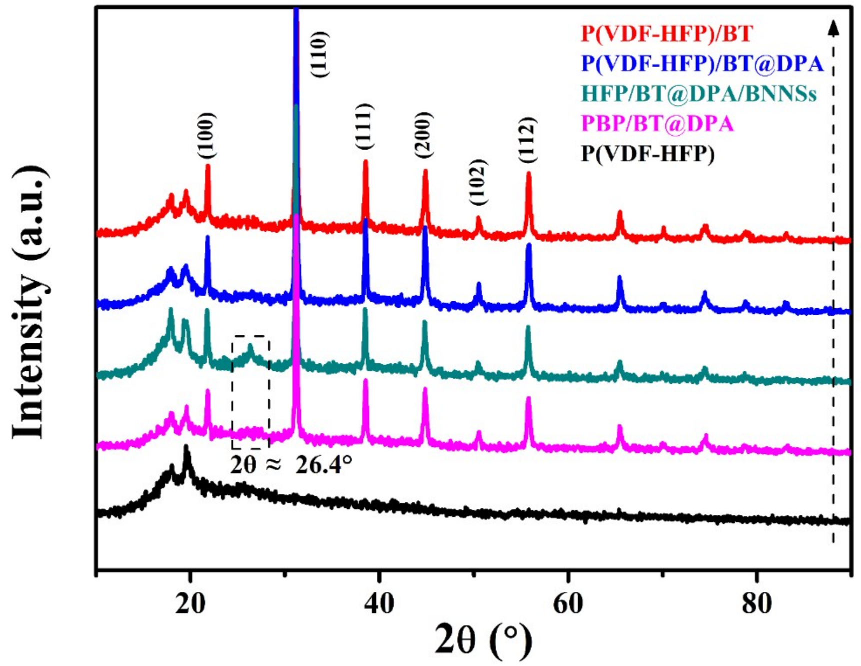

Figure S2 XRD image of several P(VDF-HFP) films

3. Weibull distribution

The films doped with different concentrations of BNNSs were analyzed for their breakdown characteristics by Weibull distribution. The result is shown in Figure S3.

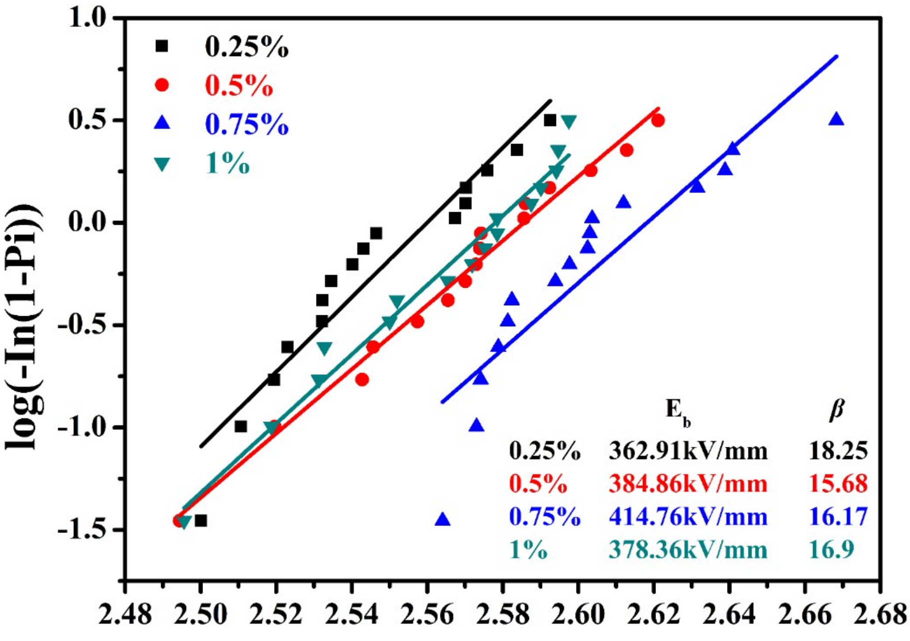

Figure S3 Weibull distribution of BNNSs concentration test

4. P-E hysteresis loops

The P-E hysteresis loops can be used to calculate the charge-discharge efficiency of ferroelectric materials. P-E hysteresis loops of films were measured by using a ferroelectric tester. Several sample films were measured under 500, 1000, 1500, 2000, 2500 and 3000 kV/cm electric fields at 10 Hz, respectively. The measurement results are shown in Figure S4.

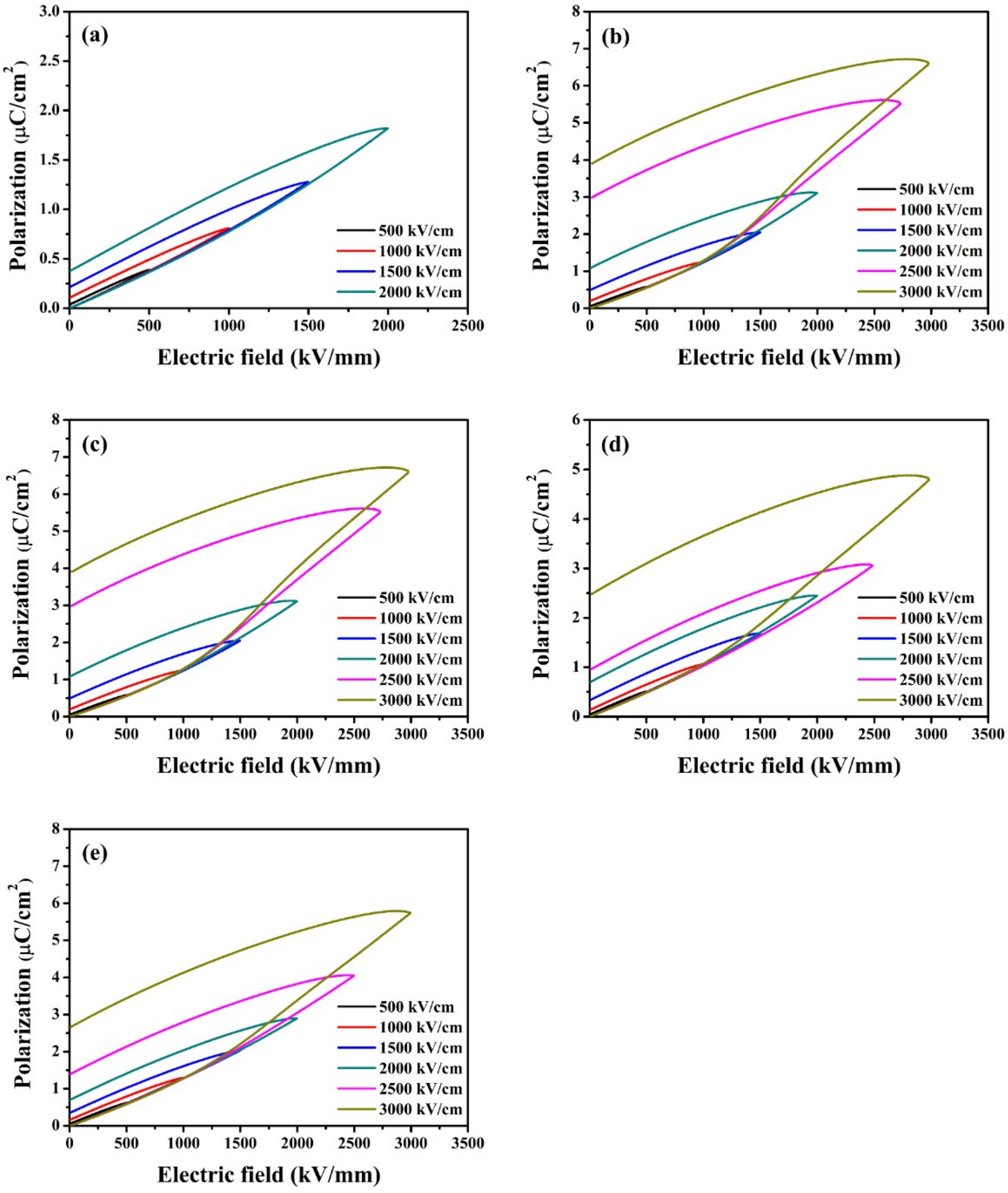

Figure S4 P-E hysteresis loops of (a) P(VDF-HFP), (b) P(VDF-HFP)/BT, (c) P(VDF-HFP)/BT@DPA, (d) HFP/BT@DPA/BNNSs, (e) PBP/BT@DPA

5. Leakage currents

As shown in Figure S5, the leakage currents of films were measured under 200, 400, 600 and 800 kV/cm electric fields, respectively. The measure time was set as 200 ms and 20000 ms, respectively.

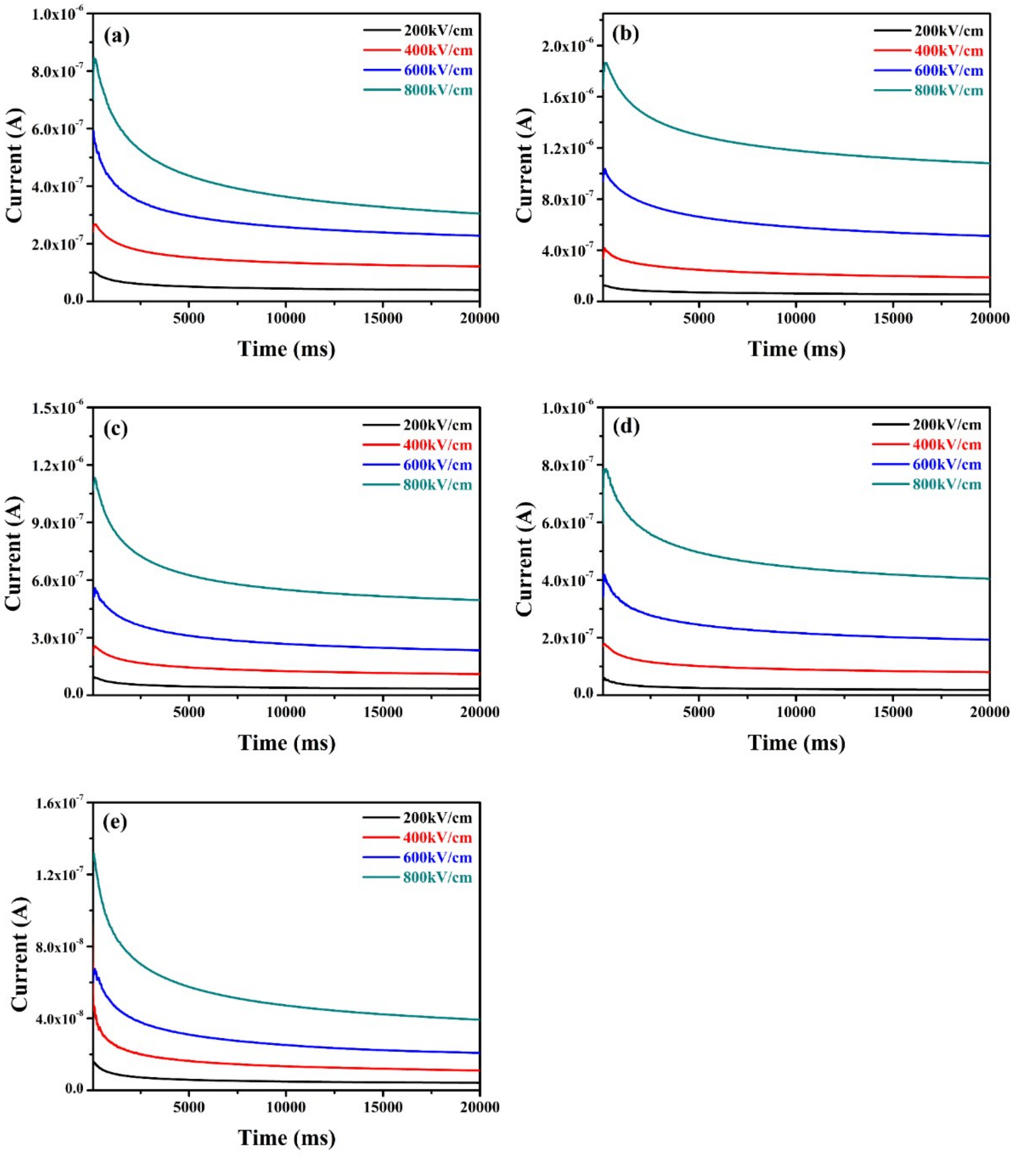

Figure S5 Leakage currents of several films from (a) P(VDF-HFP), (b) P(VDF-HFP)/BT, (c) P(VDF-HFP)/BT@DPA, (d) HFP/BT@DPA/BNNSs, (e) PBP/BT@DPA
